# Advancing gender equality through the Athena SWAN Charter for Women in Science: an exploratory study of women’s and men’s perceptions

**DOI:** 10.1186/s12961-017-0177-9

**Published:** 2017-02-21

**Authors:** Pavel V. Ovseiko, Alison Chapple, Laurel D. Edmunds, Sue Ziebland

**Affiliations:** 1 Radcliffe Department of Medicine, University of Oxford, John Radcliffe Hospital, Oxford, OX3 9DU United Kingdom; 2 Nuffield Department of Primary Care Health Sciences, University of Oxford, Radcliffe Observatory Quarter, Woodstock Road, Oxford, OX2 6GG United Kingdom

**Keywords:** Gender equality, Athena SWAN, Health research, Science policy

## Abstract

**Background:**

While in the United Kingdom, Ireland, and Australia, higher education and research institutions are widely engaged with the Athena SWAN Charter for Women in Science to advance gender equality, empirical research on this process and its impact is rare. This study combined two data sets (free- text comments from a survey and qualitative interviews) to explore the range of experiences and perceptions of participation in Athena SWAN in medical science departments of a research-intensive university in Oxford, United Kingdom.

**Methods:**

The study is based on the secondary analysis of data from two projects: 59 respondents to an anonymous online survey (42 women, 17 men) provided relevant free-text comments and, separately, 37 women participated in face-to-face narrative interviews. Free-text survey comments and narrative interviews were analysed thematically using constant comparison.

**Results:**

Both women and men said that participation in Athena SWAN had brought about important structural and cultural changes, including increased support for women’s careers, greater appreciation of caring responsibilities, and efforts to challenge discrimination and bias. Many said that these positive changes would not have happened without linkage of Athena SWAN to government research funding, while others thought there were unintended consequences. Concerns about the programme design and implementation included a perception that Athena SWAN has limited ability to address longstanding and entrenched power and pay imbalances, persisting lack of work-life balance in academic medicine, questions about the sustainability of positive changes, belief that achieving the award could become an end in itself, resentment about perceived positive discrimination, and perceptions that further structural and cultural changes were needed in the university and wider society.

**Conclusions:**

The findings from this study suggest that Athena SWAN has a positive impact in advancing gender equality, but there may be limits to how much it can improve gender equality without wider institutional and societal changes. To address the fundamental causes of gender inequality would require cultural change and welfare state policies incentivising men to increase their participation in unpaid work in the family, which is beyond the scope of higher education and research policy.

**Electronic supplementary material:**

The online version of this article (doi:10.1186/s12961-017-0177-9) contains supplementary material, which is available to authorized users.

## Background

Women are underrepresented in academic medical sciences, especially in senior leadership roles [1–5]. Participation in gender equality award schemes can be an effective means to advance gender equality [6–9]. Such schemes provide organisational impetus and motivation for positive change, serve as a framework for gender equality activities, allow sharing of good practice, generate a positive external image, and can be viewed by research funders as cost-effective because they can be implemented nationally across many institutions [8].

In the United Kingdom, the Athena Project and the Scientific Women’s Academic Network (SWAN) were set up in the early 2000s and subsequently evolved into the Athena SWAN Charter for Women in Science [10]. It has become a common and increasingly influential means to advance gender equality and has been explicitly linked to public research funding.  In July 2011, Sally Davies, the Chief Medical Officer for England, announced that applicants could not expect to be eligible for National Institute for Health Research (NIHR) Biomedical Research Centre funding “*where the academic partner* (*generally the Medical School/Faculty of Medicine*) *has not achieved at least the Silver Award of the Athena SWAN Charter for Women in Science*” [11]. According to the Equality Challenge Unit, which manages the Athena SWAN Charter, “*the proportion of Athena SWAN applications that have been from medical and medical-related departments has increased from 7.7% to 29.7% since Sally Davies’ announcement in 2011; that is a significant increase of almost 400%, which is unparalleled by the relatively stable proportion of applications from other STEMM fields encompassed such as engineering*” (Gilligan RE, Personal communication).  In May 2015, the Athena SWAN Charter broadened its scope to include arts, humanities, social sciences, business and law, professional and support staff, transgender staff and students, and men, where appropriate [12].

The Athena SWAN Charter encourages and recognises commitment to advance women’s careers in higher education and research in four key areas [13], namely representation, progression of students into academia, journey through career milestones and working environment for all staff.

Athena SWAN is based on 10 key principles (Box 1) [12]. There are three levels of awards (Bronze, Silver and Gold), for which member institutions and departments within these institutions can apply. An entry-level Bronze institution award requires an assessment of gender equality, a 4-year action plan, and an organisational structure to implement the proposed actions [14]. Department awards recognise that, in addition to institution-wide policies and actions, the department has identified particular challenges and is planning activities for the future. A Silver department award recognises that the department has successfully implemented the previously proposed actions and can demonstrate their impact [14]. Peer review panels (comprised of academics, human resources or equality and diversity practitioners, and subject specialists) assess applications, make recommendations on awards, and provide applicants with constructive feedback [15]. As more information on the effectiveness and impact of the Athena SWAN Charter becomes available [7–9, 16, 17], its membership is growing in the United Kingdom and expanding to other countries, such as Ireland and Australia. As of January 2017, there were 143 Athena SWAN members in the United Kingdom, holding 617 awards between them [18], and three Athena SWAN members in Ireland, holding eight awards between them [19]. In Australia, a total of 40 institutions – including 30 of 43 Australian universities, six medical research institutes, and four government science organisations – are working towards an Athena SWAN Bronze institution award as part of the Science in Australia Gender Equity (SAGE) pilot run by the Australian Academy of Science in partnership with the Academy of Technology and Engineering (https://www.sciencegenderequity.org.au).

**Table:** 

Box 1 Key principles of the Athena SWAN Charter [12]
1. We acknowledge that academia cannot reach its full potential unless it can benefit from the talents of all.
2. We commit to advancing gender equality in academia, in particular, addressing the loss of women across the career pipeline and the absence of women from senior academic, professional and support roles.
3. We commit to addressing unequal gender representation across academic disciplines and professional and support functions. In this we recognise disciplinary differences, including: • the relative underrepresentation of women in senior roles in arts, humanities, social sciences, business and law; • the particularly high loss rate of women in science, technology, engineering, mathematics and medicine.
4. We commit to tackling the gender pay gap.
5. We commit to removing the obstacles faced by women, in particular, at major points of career development and progression including the transition from PhD into a sustainable academic career.
6. We commit to addressing the negative consequences of using short-term contracts for the retention and progression of staff in academia, particularly women.
7. We commit to tackling the discriminatory treatment often experienced by transgender people.
8. We acknowledge that advancing gender equality demands commitment and action from all levels of the organisation and in particular active leadership from those in senior roles.
9. We commit to making and mainstreaming sustainable structural and cultural changes to advance gender equality, recognising that initiatives and actions that support individuals alone will not sufficiently advance equality.
10. All individuals have identities shaped by several different factors. We commit to considering the intersection of gender and other factors wherever possible.

While in the United Kingdom, Ireland, and Australia, higher education and research institutions are widely engaged with the Athena SWAN Charter to advance gender equality, empirical research on this process and its impact is rare. An independent mixed-methods study commissioned by the Equality Challenge Unit found that “*career satisfaction, opportunities for training and development, knowledge of promotion processes and fairness in the allocation of workload was considered better in the Silver award and other Athena SWAN category groups than in no award departments*” [7]. This study also found that the linkage to NIHR research funding was viewed contentiously, respondents suggested that women had benefited from Athena SWAN more than men, and that research staff and students had experienced less positive impact than tenured academic staff. Importantly, this study focused on the perceptions of impact rather than actual impact. A quantitative study that focused on the actual impact of Athena SWAN found no evidence that introduction of Athena SWAN in 2005 or its linkage to NIHR funding in 2011 had led to a measurable improvement in the employment rates of female clinical academics [16]. However, this study focused only on one quantitative indicator of impact (employment rate), examined only 2 years (2012–2013) after the linkage to NIHR funding, and might have been constrained by the insufficient number of observations. A multimethod qualitative realist evaluation conducted in five departments of one medical school found overwhelmingly positive perceptions of the principles of Athena SWAN [9]. Implementation was credited for creating the “*social space to address gender inequity*”, but a number of factors had reduced its impact [9]. In particular, the study found that women bore “*a disproportionate burden of Athena SWAN work, which was not counted towards career progression*”, there were barriers for postdoctoral researchers and some other staff to access Athena SWAN initiatives, and that institutional practices, national policies, and societal norms could limit the impact of Athena SWAN [9].

## Methods

### Study aim and objectives

The aim of this study is to explore the range of experiences and perceptions of participation in Athena SWAN in medical science departments at one research-intensive university in Oxford, United Kingdom. The objectives of the study are as follows:

- To explore the range of perceptions on how Athena SWAN impacts on the work conditions and employment prospects.
- To explore the range of perceptions on the implementation of Athena SWAN and how it can be improved.
- To identify key themes and issues for future research.

### Study setting

The University of Oxford is one of the United Kingdom’s most research-intensive universities and scores highly on measures of research income and performance in clinical, pre-clinical and health subjects [20, 21]. Advancing gender equality is a key strategic priority for the university [22, 23]. During the study period, all of the university’s 16 clinical and pre-clinical departments [24] participated in the Athena SWAN Charter. As of September 2016, they had achieved 15 Silver and 1 Bronze awards.

### Study design

The study is based on the secondary analysis of data from two research projects supported by the University of Oxford Vice-Chancellor’s Diversity Fund [25]. One project included administration of a quantitative C-Change survey (CCS) to measure 12 dimensions of culture [26], with additional open-ended questions. Athena SWAN was not a specific question mentioned in the survey, which focused on organisational culture more broadly [27]. The other project used face-to-face narrative interviews to build an online repository of the experiences of women in science (WIS) at Oxford (http://www.womeninscience.ox.ac.uk). This article combines data from the free-text survey comments with analysis from the narrative interviews to explore the range of experiences and perceptions of participating in Athena SWAN.

### Sample and data collection

All 3824 academic, research, administrative, professional, and other support staff in medical sciences departments at the University of Oxford on grade 6 and above were eligible to participate in the survey. The survey was conducted from May to June 2014. An email was sent by the Head of the Medical Sciences Division to all staff informing them about the survey. All eligible participants received an email, via SurveyMonkey®, with a link to the anonymous online survey. Those who had not yet completed the survey received up to 10 automated reminders over the following 6 weeks. The survey consisted mainly of closed-ended questions with four open-ended questions prompting respondents to raise new issues regarding career advancement and diversity. Athena SWAN was not mentioned in the survey. Altogether, 2407 staff responded to the survey (63%), 523 (22% of the respondents) provided free-text comments, and 59 (2% of the respondents and 11% of those who provided free-text comments) commented about Athena SWAN. Other comments concerned the role of sex, race/ethnicity, sexual orientation, disability and caring responsibilities on advancement, gender equity and underrepresentation in medicine minority faculty, and institutional change efforts for diversity and faculty support more broadly.

A purposive sample of senior women scientists working in the Medical Sciences Division (MSD) and Mathematical, Physical and Life Sciences Division (MPLS) participated in a narrative interview about their careers in October 2014 to June 2015. We wanted to interview successful women scientists from a range of departments and disciplines, so we obtained suggestions from members of the project advisory group. Information about the study was sent to 51 women. Twelve either did not reply or said that they were too busy to take part in the research. For the purpose of the current article focusing on medical sciences we excluded two interviews from MPLS.

The interviews were conducted by one of the authors (AC), who is a senior qualitative researcher with a background in medical sociology. Almost all interviews took place in the interviewee’s office. The remainder were conducted in either the woman’s home or in The Department of Primary Care Health Sciences. The interviewer did not personally know any of the women before the interview. All of the interviews were audio-taped and most were video-recorded. Interviews were conducted in private; no one else was present besides the participant and the researcher. Participants were given the option of having audio-taped interviews, instead of videos, so that they could retain anonymity. They were also given the option of making sub-sections of their video interview anonymous. Participants were informed of the study objectives as well as the interviewer’s occupation and credentials. Transcripts were returned to the participants for comment and/or correction and for copyright approval.

### Data analysis

Relevant free-text survey comments were analysed by LDE and PVO and the fully-transcribed narrative interviews by AC and SZ. Although administrative, professional and support staff had not been the focus of Athena SWAN before May 2015, we included their responses in analysis because our aim was to explore a wide range of views of the implementation of Athena SWAN, drawing on the unique perspectives of all staff in the departments. Data from the narrative interviews and open-ended questions were coded separately by the researchers from the relevant teams. The data from the open-ended questions were initially coded in Microsoft^®^ Office; NUD*IST software was used to organise the interview data. Researchers were sensitised by theories and concepts from their previous research [4, 5, 27, 28] and reviewed relevant studies during analysis to check emerging themes against the literature. We used thematic analysis and the process of constant comparison to synthesise and interpret the findings [29, 30]. The similarities and differences between the themes emerging from two datasets, were combined into a common coding tree, while preserving information about the source of the findings; for example, each quote from the data includes an ID which specifies whether it was from the CCS or WIS dataset. Researchers then met to discuss areas of agreement and disagreement and reached consensus on themes and interpretation. Researchers shared and reflected on their own prior views and experiences, which may have influenced the analysis and interpretation of data. Most notably, researchers were all members of the University, had prior knowledge of Athena SWAN and had participated in some of the related activities.

## Results

**Fig. 1:**
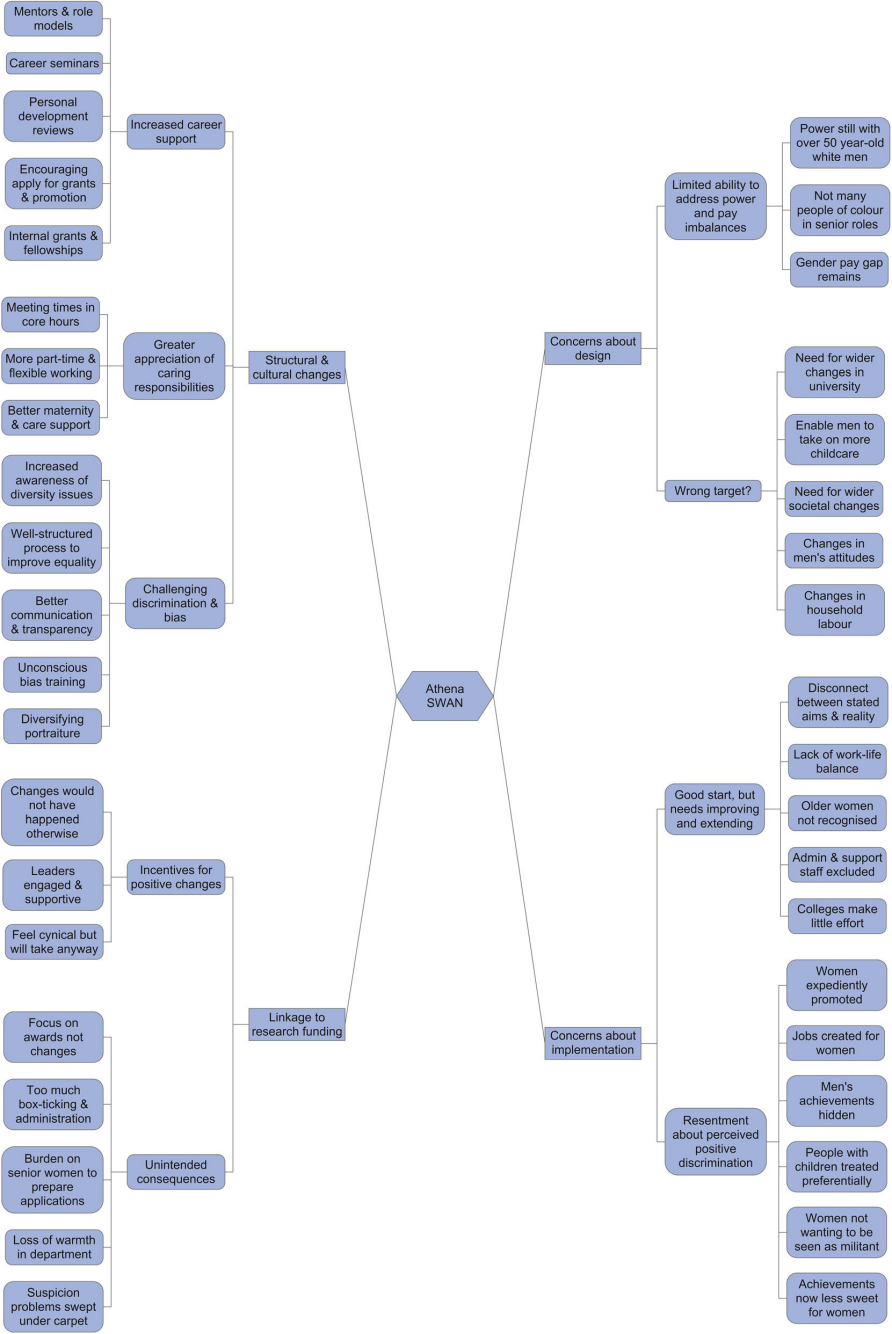
Coding tree with major themes and sub-themes

In total, 59 respondents (42 women and 17 men) wrote comments about Athena SWAN on the survey, and 37 women participated in narrative interviews. All interviewees talked about Athena SWAN (either raising it themselves in the open narrative or in response to specific questions). Interviews were, on average, an hour in length. The aggregated and disaggregated demographic characteristics of anonymous study participants are presented in Table 1 and Additional file 1, respectively. Owing to the anonymity of the survey, we do not know whether some study participants provided both free-text comments and participated in interviews. The findings are grouped into four major themes and nine sub-themes (Fig. 1) and presented below, together with illustrative quotes. Illustrative quotes are presented together with relevant demographic data for the individual, and the source of the data (CCS or WIS).

**Table 1:** Aggregated demographic characteristics of participants from each study

Characteristics	CCS Survey, n (%)	WIS Interviews, n (%)
Sex		
Female	42 (71%)	37 (100%)
Male	17 (29%)	0
Race/Ethnicity		
Black and minority ethnic	8 (14%)	6 (16%)
White	51 (86%)	31 (84%)
Staff category		
Clinical academic/research	10 (17%)	14 (38%)
Non-clinical academic/research	30 (51%)	22 (59%)
Administrative/professional/support	18 (31%)	1 (3%)
Missing data	1 (2%)	0
Full professor		
Yes	7 (12%)	20 (54%)
No	52 (88%)	17 (46%)

### Structural and cultural changes

#### Increased career support

Interview participants spoke enthusiastically about the changes associated with Athena SWAN, such as new mentoring schemes, events to increase the visibility of female role models, career development seminars and talks about women in science. In their opinion, Athena SWAN has accelerated the introduction of annual personal development reviews for all staff in the University. One senior woman explained why annual reviews were helpful:

“*…just to have an opportunity to sit down and talk about your career, not the data and the science and to get through some of people’s anxieties and to let them know and give them positive feedback because often we don’t do enough of that either…*” (WIS interview ID20, F)

According to interviewees and survey respondents, women, especially in early career stages, have been encouraged to apply for grants, fellowships and promotion due to Athena SWAN. Likewise, some departments have introduced internal pump-priming grants for early career researchers who want to take the first step towards independence and transitional fellowships for those who are not yet ready to apply for external fellowships.

#### Greater appreciation of caring responsibilities

Athena SWAN activities have led to core hours policies for meetings and seminars in the institution studied. This benefits people with childcare responsibilities without requiring them to “*make a big fuss*”, as this woman explained:

“…*we have management team meetings within our department and these are the meetings where important decisions are taken about the department, and they used to happen at five o’clock in the evening. That didn’t use to be a problem but when my daughter started school and her pick up time changed (…) I knew that wasn’t going to be sustainable. I didn’t have to make a big fuss about anything because at the same time Athena SWAN was saying that meetings had to happen in core hours. So I benefitted from the change that was taken with having that meeting at lunchtimes*.” (WIS interview ID23, F)

Other changes perceived to have occurred as a result of Athena SWAN included improved maternity leave arrangements, help with parking fees during pregnancy, subsidised nursery places, and a ‘Returning Carers’ Fund’ to support the research careers of staff returning to work after a break to care for a child or relative. In some departments, the working environment for people with caring responsibilities was perceived to have been further improved by the adoption of policies to support more flexible and part-time working for all staff.

Our survey free-text comments and interview data suggest departmental differences in perspective on Athena SWAN, which in part reflects differences between departments. For example, whereas in some departments Athena SWAN was perceived as “*box ticking exercises*”, this woman stressed that, in her department, there was a commitment to improve the situation. She went on to say:

“*I think a lot has changed in terms of culture in the department over the last two to three years* […] *There’s a general agreement that we as a department should actively try to improve the situation, not only for women, also for families, for people with caring responsibilities*.” (WIS interview ID05, F)

#### Challenging discrimination and bias

Athena SWAN was considered by responders to have increased awareness of gender and other diversity issues and provided a well-structured process to improve equality with an incentive and reward system for addressing them.

“*Athena SWAN has raised the profile of the fight against discrimination. There has been a significant backlash, but I guess it is natural to have some negative reaction from the people who don't see that the University is anything other than perfect under their leadership. ‘Who needs more women, we are doing a great job without them?’ is what comes across. It is good that the leadership is taking these issues seriously and over a few years the system will change gradually, so it is worth continuing to encourage equality*.” (CCS survey ID23, F)

Several interviewees mentioned efforts to improve communication and improve transparency on decision-making in departments. Participation in Athena SWAN was reported to have resulted in many voluntary (and some mandatory) training programmes and courses for staff as well as efforts to provide a more appropriate gender balance in portraits, or replace portraits with other images:

“[in XXX department] *there are these old Victorian type pictures of men who it now turns out have absolutely nothing to do with physiology or anything, and so we’re having a competition in the department and the students are going to put forward* [suggestions]… *not necessarily… to replace the men with women, but … to put up scientific images, rather than pictures of men*…” (WIS interview ID06, F)

### Linkage to research funding

#### Incentives for positive changes

Several interviewees and survey respondents suggested that the positive changes associated with Athena SWAN would not have happened without the government policy linking Athena SWAN awards to NIHR research funding. Many felt that this incentive had been vital:

“*To me, when it comes down to it, sure it would be great if everything happened because everyone just thought it was wonderful. But if it’s not going to happen that way and it’s something that’s going to benefit people then if it happens through a political instrument, great*.” (WIS interview ID01, F)

Participants perceived that the linkage to research funding had motivated institutional leaders to be engaged in achieving Athena SWAN awards. Respondents also sometimes stressed the importance of maintaining incentives and resources for Athena SWAN activities and the sustainability of the positive changes after the required Silver level awards were made.

“*It is my view that much of the enthusiasm for achieving Athena SWAN silver status at Oxford University is economic (i.e. concern about not being able to get NIHR grants) rather than about the loss of opportunity for women in academic careers, and that the changes may not "stick" if the financial incentive is removed*.” (CCS survey ID14, M)

#### Unintended consequences

Some respondents felt that the linkage to research funding has created perverse incentives to achieve awards rather than fundamental structural and cultural changes. Interviewees suggested that in the process of achieving awards there has been a loss of ‘warmth’ in their department due to the need to document policies for a formal Athena SWAN process, which contrasted with the previous responsive, informal culture. The application process was seen as involving too much ‘box-ticking’ and administration:

“*I’m a bit cynical about Athena SWAN. I think it started off as a really good thing but the problem now is that it’s been tied to funding* […] *The department has to have Athena SWAN accreditation. And that means if it has to be got then it will be got by whatever means. And I think that then turns what could be quite a useful exercise into a box-ticking exercise. So I think it creates a huge amount of administration in the department*.” (WIS interview ID09, F)

Others felt that senior women are now bearing the burden of preparing Athena SWAN applications and spending time on too many committees.

“*Since I became a PI* [principal investigator] *in the department, there is a lot of this Athena SWAN activity.* […] *They are kind of desperate to get the few female senior academics that are around on committees* […] *some people feel really stretched because they are asked to do lots of committees, panels, because it’s always good not to have a male only panel, and, hopefully, this will change over the years, when kind of more female researchers will grow up and get in to more senior posts*.” (WIS interview ID05, F)

There were suspicions that some problems were swept under the carpet to avoid jeopardising the award application process. Some participants resented changes that they saw as incidental or ‘tokenistic’, citing a brunch for women or professorial titles (for example, through the recognition of distinction exercise) without changes to duties or salaries. However, more substantial changes, such as appointing women to new posts, were also sometimes regarded with suspicion:

“*My department recruited two new investigators this last year, both of whom were female. It seemed this was due to meeting an Athena SWAN goal (as before Athena SWAN application all PIs were male) rather than including them for their capability*.” (CCS survey ID55, F)

### Concerns about Athena SWAN design

#### Limited ability to address power and pay imbalances

A number of survey respondents and interview participants were concerned about the limited ability of Athena SWAN to address longstanding and entrenched power imbalances by gender, age and ethnicity.

“*Much talk about supporting women through the Athena SWAN initiatives but power in the department still held by white men aged 50+*.” (CCS survey ID03, F)

“*There isn't a single female head of department. The head of* [XXX department] *is actively giving women the title of associate professor as a tokenistic way of getting his Athena SWAN badge. Where are the people of colour? I certainly don't see many of them in senior roles. I again can't think of one that's a head of department*…” (CCS survey ID30, F)

Another important concern expressed by survey respondents related to the perceived pay imbalances. Although institutions applying for Athena SWAN awards could choose to address pay imbalances as part of their action plans, this had not (yet) been the case in this setting. One survey respondent suggested examining pay imbalances:

“*There are many very talented and able women in the University, and they do succeed, but they are expected to achieve more and they receive less. It would be very interesting to do a direct side-by-side comparison of salary versus workload. Athena SWAN does not address the issue of pay. It would be interesting to compare. I am sure that the imbalance is even worse when it comes to ethnic minorities. The male/female split has been addressed to some extent by Athena SWAN, but there is no similar organisation looking at ethnicity, gender orientation, age, or responsibilities*.” (CCS survey ID23, F)

#### Wrong target?

Women and men raised concerns that achieving an Athena SWAN Silver award might be the wrong target as attempts to increase gender equality at work were unlikely to succeed without wider cultural and structural changes in the university as well as society. A female administrator commented in the survey about the need to change the structure of employment within the university given that the high proportion of short-term grant-funded projects adversely affected the well-being, work-life balance, and overall career progression of staff on fixed-term contracts linked to grant-funded projects. Some raised concerns that Athena SWAN activities were in danger of tinkering around the margins of a much greater problem of gender inequality in society, especially in relation to child rearing. Wider societal changes were needed, including changes in men’s attitudes and in the division of household labour.

“*The solution to the problem of inequality at the top level is not positive discrimination, it must be to create a society that encourages men to do their fair share of the domestic work. This will not be done by the current system*.” (CCS survey ID01, M)

To facilitate these changes, Athena SWAN needs to provide more support for men as equal contributors to childcare:

“*If the university really wants to support women and promote family friendly working practices then they have to treat men as serious contributors to childcare and support them accordingly. Gender inequality isn't just about what happens to women, what happens to men is crucial too.*” (CCS survey ID38, F)

### Concerns about Athena SWAN implementation

#### Good start, but needs improving and extending

The positive changes perceived to have been brought about by Athena SWAN were largely welcomed (especially by women), but some respondents expressed concerns about its implementation and exclusion of particular groups of staff. Some perceived a disconnect between the stated aims of Athena SWAN and reality in their own department, especially with respect to persisting lack of work-life balance in academic medicine:

“*I think Athena Swan is beginning to impact but there is still a big difference between what is stated as an ideal practice and what actually happens on the ground. For I was present at an Athena SWAN meeting when it was stated that the department needed to be more aware of the hours people work and a Professor was very positive in his support for that but when the chips are down he still expects people to be available at evenings and weekends and I am personally aware that some of his staff (male!) have been working 60-hour weeks for two years or more with no respite and none yet on the horizon. This is where subconscious discrimination gets a hold – very few women are able to support that type of commitment/lifestyle and the staff mentioned above can only do so because they are supported fully by their wives who run the family for them*.” (CCS survey ID50, F)

Other survey respondents raised concerns that departments excessively focussed on the appointment and promotion of staff with caring responsibilities at the expense of older women. Others noted that, in the early rounds (see Discussion) the administrative, and other support staff had been excluded from Athena SWAN activities.

“*Athena SWAN has kick-started the advancement of women in the university. However, it is really focussed on researchers. What about all the academic support staff who support, promote, advise, help to manage the entire research enterprise? I 100% support the drive to improve the promotion of women in academia, I'm just baffled as to how it can exclude all the other women working with academics*.” (CCS survey ID27, F)

Oxford is a collegiate university consisting of academic departments and self-governing colleges. The latter provide fellows with the opportunity to be part of an interdisciplinary scholarly community, take part in its governance, access college research funds and benefits, mentor and select new students and fellows. Unlike departments, colleges do not currently participate in Athena SWAN. One survey respondent felt that the colleges, where many of the activities take place in the evening, were lagging behind departments in their efforts to support diversity and needed to change.

“*The Athena SWAN scheme is making great advances towards equality and fairness within the departments. However, colleges are still very hostile places for minorities, women and those with children. I am a fellow at one of the university colleges, but find that the college makes very little effort to adapt its practices to enable a female, from a minority and with childcare responsibilities, like myself, to take an active part in college life*.” (CCS survey ID08, F)

#### Resentment about perceived positive discrimination

Survey responses indicated concerns about perceived positive discrimination. A number of respondents (almost all men) who commented in the survey suggested that women were now being expediently promoted, new jobs were being created especially for women, and men’s achievements were being hidden.

“*I am a passionate advocate of equality but we are now in a period of preferment and this goes against the principle of equality. It is unacceptable and generating substantial resentment that is difficult to express openly. Many senior colleagues have confided similar views to me*.” (CCS survey ID01, M)

“*I see those around me openly discriminating based on gender and ethnicity and that it is celebrated with awards, such as the Athena SWAN award. I have been asked to hide the successes of men from our websites and to make specific changes to the sites to encourage anyone other than white men to apply for jobs*.” (CCS survey ID12, M)

Another suggested that people with children are now treated preferentially at the expense of those without.

“*Things like Athena SWAN ensure that people with young families are treated better at the expense of people who do not have children – for example, seminars rescheduled to fit in with school runs at the expense of those who have other things to do with their time*.” (CCS survey ID10, M)

There were also women who did not want to have their careers defined by Athena SWAN and who did not want to be perceived as ‘militant’. A successful woman scientist felt that, inadvertently, the perception of ‘quotas’ might diminish women’s achievements.

“*I think Athena SWAN has definitely changed the landscape. Is it for the better? In some ways ‘yes,’ in some ways ‘no’. Before, if I got somewhere, I got somewhere on my own merit because I did something. Now you might say, ‘Am I getting somewhere because they need a ‘quota woman’ or they need to have more senior women?’… and it makes the achievements slightly less sweet, if that makes sense.*” (WIS interview ID 02, F)

## Discussion

There are various controversial ‘explanations’ for gender inequality in science, including that women may not want high- powered jobs, that women have different innate ‘aptitudes’ and ‘attributes’ to men, and that women in science face discrimination and stereotyping [31–34]. There is some consensus among social scientists that negative stereotyping has deterred women from choosing science as a career, and at least historically, has also impaired women’s careers in science [35]. Our study contributes to the emerging literature on gender equality award schemes by extending the existing evidence base on the impacts of Athena SWAN. A comparison of findings from our combined analysis with those from the mixed-methods multi-centre evaluation by Munir et al. [7] and the most recent single-centre qualitative realist evaluation by Caffrey et al. [9] suggests considerable convergence in perceptions about the positive impact of Athena SWAN. Recurrent across the studies are themes regarding mentoring, leadership and career development, performance review and promotions, flexible working, maternity and childcare arrangements, core hours and other family-friendly policies, and unconscious bias training. Our findings support Caffrey et al.’s conclusion that the implementation of Athena SWAN had created “*social space to address gender inequity*” [9]. In addition, our study highlights initiatives that may serve as examples of good institutional practice, e.g. new internal pump-priming grants and transitional fellowships for early career researchers, a Returning Carers’ Fund to support researchers returning to work after a break due to caring responsibilities, and efforts to diversify the working environment by improving gender balance in portraits.

Notwithstanding its perceived positive impact, our findings also reveal important concerns about Athena SWAN. Unlike Munir et al. [7], who found considerable evidence that the positive changes associated with Athena SWAN were sustainable, we found concerns about the sustainability of the positive changes once the required Silver level award is achieved. Moreover, we found concerns about Athena SWAN’s limited ability to address longstanding and entrenched power and pay imbalances, persisting lack of work-life balance in academic medicine, as well as a concern that achieving the award could become an end in itself, and resentment about perceived positive discrimination. Our findings support the finding by Caffrey at al. [9] that women were thought to bear a disproportionate burden of administrative work in Athena SWAN initiatives, but we are unable to ascertain how this might impact their career advancement and leadership. On the one hand, this might have a negative impact on women’s research productivity and thus their advancement and leadership because “*research is the pathway to leadership in academic environments*” [36]. On the other hand, participation in Athena SWAN committees and administration may support women’s advancement and leadership. In the given institution, service on Athena SWAN committees and related administrative work count towards ‘good citizenship’, which in addition to research and teaching is a compulsory criterion for academic appointments and promotions [37]. Likewise, there is evidence from United States and Canadian medical school deans that appointing women to high-level committees and task forces supports women’s advancement and leadership [38].

Our study provides some insights into the role of financial incentives. Although work to support equality was on going (one interviewee recalled being involved in Athena SWAN in the early 2000s when it was established), substantial activity in pursuit of Athena SWAN awards in medical science departments in the given institution began in 2011, when NIHR funding was linked to Silver awards. Similar to the findings of Munir et al. [7] and Caffrey et al. [9], many of our study participants thought that the positive changes associated with Athena SWAN would not have taken place without the linkage. Some perceived unintended consequences, such as the perceived focus on awards rather than changes, an administrative burden, especially on senior women, and the perverse incentive to deny problems to pass the assessment. The focus of our study on the single institution brings to the fore concerns about programme implementation at the institutional level versus programme design at the national level. Athena SWAN is based on the permissive approach, according to which participating institutions decide themselves which measures and policies to implement. Therefore, many of the concerns raised in the survey and interviews relate to the implementation of Athena SWAN in the given institution and thus need to be addressed at the institutional level. For example, it is important to investigate whether the perceived instances of positive discrimination reported by the participants referred to positive action or indeed positive discrimination. Following the enactment of the United Kingdom Equality Act 2010, positive action (i.e. hiring and promoting candidates from under-represented groups who are as qualified as the other candidates) has become lawful, but positive discrimination (hiring and promoting candidates just because they are from the under-represented groups) remains unlawful.

In the early rounds, Athena SWAN focused exclusively on academic and research staff. In the period when this study was conducted, professional and support staff were involved in activities concerning all staff, such as the surveys of departmental culture or brunches for women, but not as the intended beneficiaries of the core resource-intensive policies, e.g. the Returning Carers’ Fund. Likewise, in the period under investigation, Athena SWAN focused exclusively on women. In May 2015, when data collection for this study was almost complete, Athena SWAN broadened its scope to focus also on professional and support staff, transgender staff and men, where appropriate. Moreover, Oxford and other collegiate universities are working with the Equality Challenge Unit to extend Athena SWAN to colleges as well. It remains to be seen how these changes to Athena SWAN design will affect its implementation at the institutional level.

Our findings also reveal concerns that can only be addressed with policies to promote cultural changes at the national level. Both Caffrey et al.’s study [9] and ours reported perceptions that, in order to achieve a greater degree of gender equality, further structural and cultural changes are needed at all levels of the university and in wider society. These perceptions are best interpreted in the context of wider social science literature. Fundamentally, gender inequality in society stems from the unequal division of paid work outside the family and unpaid work in the family [39]. Some of the most advanced Nordic welfare states have policies to incentivise men to increase their participation in unpaid work. In the Nordic countries characterised by the highest levels of gender equality, men are incentivised to take parental leave and both parents receive high levels of compensation for the loss of income while on leave. For example, Iceland provides up to 3 months of taxpayer-funded parental leave exclusively for fathers [40].

Taking into account that both institutional responses to Athena SWAN and individual choices regarding the division of paid and unpaid work in the family are influenced by societal norms and welfare state policies, there are limits to how much Athena SWAN can improve gender equality without wider institutional and societal changes. The majority of positive changes associated with the implementation of Athena SWAN – such as increased support for women’s careers, mentoring, role models, visibility of women in science, and efforts to challenge discrimination and bias – focus on making the academic science workplace a less gendered environment. Initiatives which focus on family care- giving will be counter-productive if they position the activities as supporting women, rather than parents of both genders. The implementation of Athena SWAN should provide parents of both genders with more choices to combine career advancement and family life.

### Strengths and limitations

This is one of the first studies to explore the range of perceptions of participation in Athena SWAN initiatives, which are intended to advance gender equality. The study setting provides a fertile ground for such an exploration because Athena SWAN has a high profile in medical sciences at this university.

For the analysis in this paper we have brought together two datasets, collected for other purposes, which provide new insights. Our narrative interview study captured the experiences and perspectives of a diverse sample of successful women scientists, all of whom were aware of Athena SWAN activities. The survey was anonymous to encourage honest responses from women and men across different career stages and levels of seniority. The survey questionnaire was intended to provide comparative data with overseas universities and therefore did not include specific questions about Athena SWAN initiatives. Only 2% of responders to the survey provided free-text comments on Athena SWAN. The voluntary open-ended nature of questions in the survey might represent those with strong views. Those who provided anonymous survey responses might have been more inclined to express negative attitudes [41]. The small number of free-text survey responses mentioning Athena SWAN precluded quantitative analysis. Given that our sample is not statistically representative, the range of themes and sub-themes is neither exhaustive nor statistically representative. Thus, the study increases understanding of the range of perceptions, without attempting to quantify their prevalence.

Our analysis provides new findings, some of which may be context-specific. Interviews with senior men in science would probably add further perspectives. However, the analysis of the free-text comments from the survey adds insights from staff with other roles, including men at all grade levels, to the perspectives of the senior women. We can claim some theoretical generalisability, suggesting that our findings may transfer to other settings and future research. For example, the themes identified in our study could be used to develop questions and categories for research using qualitative, survey or mixed-methods designs and provide a point of comparison for other studies with participants working in science, clinical or health sciences.

Finally, our study increases value and reduces waste in research by using and sharing the existing datasets [42]. Our study design saves the costs of surveying and interviewing the same population both in terms of new research expenses and time of participants, many of whom are clinicians. Throughout the study, we have reported participant-level data containing both the perceptions of Athena SWAN and demographic characteristics of respondents. When reporting aggregated perceptions, we have stated whether they came from the survey or interviews.

### Implications for practice and research

Given that participation in Athena SWAN may have significant impact on people’s work conditions and employment prospects, strong views about its impact are to be expected. In light of our analysis of experiences and perceptions of this programme, there is also a need to evaluate the actual impacts on both genders across all staff groups. In so doing, it will be important to take into account the influence of the confounding factors such as gender equality legislation and various university-wide activities. The latter are particularly important because Athena SWAN has provided impetus for many gender equity and diversity policies and initiatives, which should be further evaluated.

The findings provide a valuable starting point to investigate further and address the concerns of women and men about Athena SWAN initiatives and their local implementation. Comparative research into convergence and divergence of Athena SWAN implementation across different institutions may illuminate the effects of different approaches to Athena SWAN. Sustainability of the perceived positive changes in the post-award period is also a key aspect of Athena SWAN that would benefit from comparative research into Athena SWAN implementation. Despite the competitive relationships between higher education and research institutions, an Athena SWAN community of practice is developing to share good practice across institutional boundaries. The limited ability of Athena SWAN to address longstanding and entrenched power and pay imbalances may require a different approach and a longer-term view may be needed to address such imbalances. Different institutions may have different approaches to distributing the administrative burden of Athena SWAN work between women and men as well as different recognition and reward systems for their contribution to Athena SWAN initiatives, with implications for career advancement.

The perceived importance of the financial incentives for bringing about positive changes suggests that future evaluations should differentiate between research-intensive institutions and departments within institutions that can benefit the most and the least from the financial incentives. Future evaluations would benefit from mixing different methods of data collection, including in-depth interviews with men and women at all grades and anonymous surveys with more direct questions based on the themes and hypotheses from the current research and wider social science theory.

## Conclusions

This article provides insight into perceptions of Athena SWAN in one university. Those who commented on Athena SWAN widely (though not universally) suggested that it had a positive impact in advancing gender equality, but also raised concerns about the programme design and implementation. Both the findings from this study and insights from wider social science literature suggest that there may be limits to how much Athena SWAN can improve gender equality without wider institutional and societal changes. To address the fundamental causes of gender inequality would require cultural change and welfare state policies incentivising men to increase their participation in unpaid work in the family, which is beyond the scope of higher education and research policy. The findings make an original contribution to the emerging literature on gender equality award schemes by extending the existing evidence base and drawing implications for practice and research.

## Additional file

Additional file 1: Disaggregated demographic characteristics of study participants. Anonymised study participants by sex, race/ethnicity, staff category, and full professorial rank. (PDF 225 kb)

## Abbreviations

Athena SWAN: Athena Project and the Scientific Women’s Academic Network
CCS: C-Change survey
MPLS: Mathematical, Physical and Life Sciences Division
MSD: Medical Sciences Division
NIHR: National Institute for Health Research
PI: principal investigator
SAGE: Science in Australia Gender Equity
STEMM: science, technology, engineering, mathematics and medicine
WIS: women in science.
