# Supplementary material for: Advancing gender equality through the Athena SWAN Charter for Women in Science: an exploratory study of women’s and men’s perceptions
Source: Health Res Policy Syst. 2017 Feb 21;15:12. doi: 10.1186/s12961-017-0177-9 (PMC5320775; doi:10.1186/s12961-017-0177-9)
Supplement: Additional file 1: — Disaggregated demographic characteristics of study participants. Anonymised study participants by sex, race/ethnicity, staff category, and full professorial rank. (PDF 225 kb) [file 12961_2017_177_MOESM1_ESM.pdf]

Additional file 1: Disaggregated demographic characteristics of study participants

ID	Sex	Race/ Ethnicity	Staff Category	Full Professor
CCS 01	M	White	Clinical academic/research	Yes
CCS 02	F	White	Clinical academic/research	No
CCS 03	F	White	Clinical academic/research	No
CCS 04	F	White	Non-clinical academic/research	Yes
CCS 05	M	White	Non-clinical academic/research	No
CCS 06	F	White	Non-clinical academic/research	No
CCS 07	F	White	Non-clinical academic/research	No
CCS 08	F	BME	Non-clinical academic/research	No
CCS 09	M	White	Non-clinical academic/research	Yes
CCS 10	M	White	Non-clinical academic/research	No
CCS 11	M	White	Administrative/professional/support	No
CCS 12	M	White	Administrative/professional/support	No
CCS 13	M	White	Administrative/professional/support	No
CCS 14	M	White	Clinical academic/research	Yes
CCS 16	F	White	Clinical academic/research	No
CCS 17	M	BME	Non-clinical academic/research	No
CCS 18	F	White	Non-clinical academic/research	No
CCS 20	F	White	Non-clinical academic/research	No
CCS 22	F	White	Non-clinical academic/research	No
CCS 23	F	White	Non-clinical academic/research	No
CCS 24	F	White	Non-clinical academic/research	No
CCS 25	F	White	Non-clinical academic/research	No
CCS 27	F	White	Administrative/professional/support	No
CCS 28	M	BME	Administrative/professional/support	No
CCS 29	F	White	Administrative/professional/support	No
CCS 30	F	BME	Administrative/professional/support	No
CCS 31	F	White	Administrative/professional/support	No
CCS 32	M	White	Clinical academic/research	Yes
CCS 33	F	White	Clinical academic/research	No
CCS 35	F	White	Non-clinical academic/research	No
CCS 36	F	White	Non-clinical academic/research	No
CCS 37	F	White	Non-clinical academic/research	No
CCS 38	F	White	Non-clinical academic/research	No
CCS 39	M	White	Non-clinical academic/research	No
CCS 40	M	BME	Non-clinical academic/research	No
CCS 41	F	White	Non-clinical academic/research	No
CCS 42	F	White	Non-clinical academic/research	No
CCS 44	F	White	Non-clinical academic/research	Yes
CCS 45	F	White	Non-clinical academic/research	No
CCS 46	F	White	Non-clinical academic/research	No
CCS 47	F	White	Non-clinical academic/research	No
CCS 49	M	White	Administrative/professional/support	No
CCS 50	F	White	Administrative/professional/support	No
CCS 51	F	White	Administrative/professional/support	No
CCS 52	F	BME	Administrative/professional/support	No
CCS 53	F	White	Administrative/professional/support	No
CCS 54	F	White	Administrative/professional/support	No
CCS 55	F	White	Missing data	No

ID	Sex	Race/ Ethnicity	Staff Category	Full Professor
CCS 56	M	White	Clinical academic/research	No
CCS 57	F	White	Clinical academic/research	No
CCS 58	F	BME	Clinical academic/research	No
CCS 60	F	White	Non-clinical academic/research	No
CCS 61	F	White	Non-clinical academic/research	No
CCS 62	F	White	Non-clinical academic/research	No
CCS 64	M	White	Non-clinical academic/research	No
CCS 65	M	White	Administrative/professional/support	No
CCS 66	F	White	Administrative/professional/support	No
CCS 68	F	White	Administrative/professional/support	No
CCS 71	F	BME	Administrative/professional/support	No
WIS01	F	White	Non-clinical academic/research	No
WIS02	F	White	Non-clinical academic/research	Yes
WIS03	F	White	Non-clinical academic/research	Yes
WIS04	F	White	Clinical academic/research	No
WIS05	F	White	Non-clinical academic/research	No
WIS06	F	White	Non-clinical academic/research	No
WIS07	F	White	Non-clinical academic/research	No
WIS08	F	White	Clinical academic/research	Yes
WIS09	F	White	Non-clinical academic/research	No
WIS10	F	White	Non-clinical academic/research	Yes
WIS12	F	White	Non-clinical academic/research	Yes
WIS13	F	White	Non-clinical academic/research	No
WIS14	F	White	Non-clinical academic/research	No
WIS15	F	White	Non-clinical academic/research	No
WIS16	F	White	Administrative/professional/support	No
WIS17	F	BME	Non-clinical academic/research	No
WIS18	F	White	Non-clinical academic/research	Yes
WIS19	F	White	Clinical academic/research	Yes
WIS20	F	White	Non-clinical academic/research	Yes
WIS22	F	White	Clinical academic/research	Yes
WIS23	F	White	Non-clinical academic/research	Yes
WIS24	F	White	Clinical academic/research	No
WIS25	F	BME	Clinical academic/research	No
WIS26	F	White	Clinical academic/research	Yes
WIS27	F	White	Clinical academic/research	No
WIS28	F	White	Clinical academic/research	Yes
WIS30	F	BME	Non-clinical academic/research	Yes
WIS31	F	BME	Non-clinical academic/research	No
WIS32	F	White	Non-clinical academic/research	Yes
WIS33	F	BME	Non-clinical academic/research	Yes
WIS34	F	White	Non-clinical academic/research	Yes
WIS35	F	White	Clinical academic/research	Yes
WIS36	F	White	Clinical academic/research	Yes
WIS37	F	White	Clinical academic/research	Yes
WIS38	F	BME	Clinical academic/research	No
WIS39	F	White	Clinical academic/research	No
WIS40	F	White	Non-clinical academic/research	Yes
